# Draft Genome Sequences of 142 Mycobacterium avium subsp. *paratuberculosis* Strains Isolated from Naturally Infected Dairy Cattle

**DOI:** 10.1128/MRA.00697-21

**Published:** 2021-09-23

**Authors:** Cyril Conde, Maxime Branger, Thierry Cochard, Marie-Noëlle Rossignol, Christine Fourichon, Arnaud Delafosse, Aurore Davergne, Alain Joly, David Ngwa-Mbot, Laurent Journaux, Raphael Guatteo, Laurent Schibler, Franck Biet

**Affiliations:** a INRAE, Université de Tours, ISP, Nouzilly, France; b INRAE, GABI, AgroParisTech, Université Paris-Saclay, Jouy-en-Josas, France; c INRAE, UMR1300 Biologie, Epidémiologie et Analyse de Risque en Santé Animale; Centre Nantes-Angers, Nantes, France; d Groupement de Défense Sanitaire Orne, Alençon, France; e GDMA, Bois-Guillaume, France; f Groupement de Défense Sanitaire Bretagne, Vannes, France; g GDS France, Paris, France; h Idele, Paris, France; i Allice, Paris, France; Indiana University, Bloomington

## Abstract

Mycobacterium avium subsp. *paratuberculosis* is the etiological agent of Johne’s disease in ruminants. Here, we report the annotated draft genome sequences of 142 M. avium subsp. *paratuberculosis* strains that were isolated from dairy cattle in France between 2014 and 2018. The genomes of these strains were sequenced using Illumina technology.

## ANNOUNCEMENT

Mycobacterium avium subsp. *paratuberculosis* is an economically significant veterinary pathogen in cattle, sheep, and goat populations (1). M. avium subsp. *paratuberculosis* is a very clonal species with genomes characterized by a high homology (2). To study the diversity of M. avium subsp. *paratuberculosis* strains and their phylogeny, it is therefore essential to have their entire genome sequences available.

Therefore, this sequencing project was conducted to sequence a panel of 142 M. avium subsp. *paratuberculosis* strains isolated during a longitudinal study of infected bovine (3) to better define the genetic features and pangenomic characteristics of this veterinary pathogen.

The 142 M. avium subsp. *paratuberculosis* strains were isolated from fecal samples collected as part of a longitudinal study of naturally infected cattle (3). The M. avium subsp. *paratuberculosis* isolates were propagated on slopes of Middlebrook 7H11 agar, 0.5% (vol/vol) glycerol, 10% (vol/vol) Middlebrook oleic acid-albumin-dextrose-catalase (OADC) enrichment medium (Becton, Dickinson, Oxford, Oxfordshire, UK), and 2 μg/ml of mycobactin J (IdVet, France) at 37°C. The isolates were tested for the presence of the M. avium subsp. *paratuberculosis*-specific sequence IS*900* (4) and genotyped using the MLVA (4).

Mycobacteria were harvested in the early to mid-log phase of growth, and the cells were pelleted at room temperature for 10 min at 6,000 × *g*. The pellets were resuspended in 10 ml of TE buffer (10 mM Tris-HCl [pH 8.0], 1 mM EDTA) and centrifuged again at 6,000 × *g* for 10 min. The semidried mycobacterial pellets were resuspended in 360 μl of lysis buffer T1 (Macherey-Nagel, Germany, NucleoSpin tissue kit), and the samples were transferred to a Lysing Matrix B tube (mpbio) containing 350 mg of 0.1 mm silica spheres. The samples were homogenized in a FastPrep FP120 cell disruptor at 3 × 20 s, speed 6, followed by centrifugation at 14,000 × *g* for 5 min. The supernatants were transferred to fresh sterile microcentrifuge tubes, 25 μl of proteinase K (20 mg/ml) (Macherey-Nagel) was added, and the samples were incubated for 16 h at 56°C. DNA was extracted using the NucleoSpin tissue kit (Macherey-Nagel) according to the manufacturer’s protocol. DNA was quantified using the QuantiFluor double-stranded DNA (dsDNA) system with the Quantus fluorometer (Promega, France). The extracted DNA (10 to 30 ng) was used to prepare sequencing libraries with the Nextera XT kit (Illumina, San Diego, CA), and sequencing was conducted using the Illumina MiSeq instrument at the @Bridge platform (INRAE, GABI, Jouy en Josas, France). Sequencing was performed in paired-end, 300-bp format and was downsampled to about 50× coverage. The short-read data were first trimmed using fastp v0.20.1 (5), based on a Phred scale base quality threshold of Q30 and removing reads of less than 21 bp. Kraken2 v2.0.9-beta (6) with the bacteria database was used to perform taxonomic identification at the read level in order to check for potential contamination (Table 1). All reads assigned to the *Mycobacteriaceae* family were extracted and then assembled using SPAdes v3.14 (7) with the “-careful” option. The metrics of the assembled contigs were collected using QUAST v5.0.2 (8), and the contigs were annotated using Prokka v1.14.6 (9) with M. avium subsp. *paratuberculosis* K-10 as the reference annotation strain (10, 11). Prodigal (12) was pretrained using the complete genome sequence of strain K-10.

**TABLE 1:** Sequencing metrics for the 142 Mycobacterium avium subsp. paratuberculosis strains analyzed

Isolate	Genome size (bp)	No. of contigs	*N*_50_ (bp)	Total no. of genes	Total no. of CDSs^*a*^	G+C content (%)	ENA accession no.	Contig accession no.
PICSAR1	4,788,264	169	66,775	4,572	4,509	69.32	ERS6092665	CAJSNF010000001
PICSAR10	4,806,270	342	33,849	4,633	4,570	69.31	ERS6092666	CAJSMR010000001
PICSAR100	4,790,864	141	77,512	4,585	4,522	69.32	ERS6377149	CAJSMZ010000001
PICSAR102	4,793,984	158	74,709	4,582	4,519	69.32	ERS6092667	CAJPZJ020000001
PICSAR103	4,791,349	169	68,061	4,580	4,517	69.32	ERS6092668	CAJSMU010000001
PICSAR104	4,785,660	212	51,002	4,583	4,521	69.31	ERS6092669	CAJSND010000001
PICSAR106	4,784,162	168	58,265	4,568	4,505	69.32	ERS6092670	CAJSNC010000001
PICSAR107	4,783,589	205	54,247	4,597	4,534	69.32	ERS6092671	CAJSMV010000001
PICSAR11	4,786,941	216	50,211	4,602	4,540	69.31	ERS6092672	CAJSMY010000001
PICSAR110	4,785,567	188	56,293	4,586	4,523	69.31	ERS6092673	CAJSMS010000001
PICSAR111	4,780,529	170	56,292	4,572	4,509	69.32	ERS6092674	CAJSNE010000001
PICSAR113	4,784,630	180	62,923	4,579	4,516	69.31	ERS6092676	CAJSNG010000001
PICSAR117	4,788,531	167	63,942	4,566	4,503	69.32	ERS6092678	CAJSMT010000001
PICSAR118	4,780,126	206	52,735	4,595	4,532	69.31	ERS6092679	CAJSNB010000001
PICSAR119	4,786,159	196	50,347	4,577	4,514	69.31	ERS6092680	CAJSMW010000001
PICSAR120	4,788,510	204	56,691	4,589	4,526	69.3	ERS6377150	CAJSMX010000001
PICSAR124B	4,787,722	136	86,432	4,570	4,507	69.32	ERS6377151	CAJSNA010000001
PICSAR124	4,792,645	158	71,665	4,573	4,510	69.32	ERS6092682	CAJSNI010000001
PICSAR126	4,796,308	164	86,432	4,561	4,498	69.33	ERS6092684	CAJSNH010000001
PICSAR13	4,789,268	146	80,438	4,569	4,506	69.32	ERS6092687	CAJSNJ010000001
PICSAR131	4,790,689	128	92,490	4,561	4,498	69.32	ERS6377152	CAJSNU010000001
PICSAR132	4,787,497	177	63,521	4,583	4,520	69.32	ERS6092689	CAJSNQ010000001
PICSAR135	4,794,273	153	91,217	4,583	4,520	69.33	ERS6092690	CAJSNR010000001
PICSAR136	4,788,400	184	59,721	4,586	4,523	69.32	ERS6092691	CAJVAR010000001
PICSAR14	4,787,453	243	41,260	4,609	4,546	69.31	ERS6092693	CAJSNP010000001
PICSAR140	4,789,215	126	92,229	4,563	4,500	69.33	ERS6092694	CAJSNW010000001
PICSAR141	4,786,839	168	71,205	4,574	4,511	69.31	ERS6092695	CAJSNX010000001
PICSAR142	4,787,534	161	64,094	4,581	4,518	69.32	ERS6092696	CAJSNS010000001
PICSAR143	4,779,428	192	55,835	4,570	4,507	69.32	ERS6092697	CAJSNT010000001
PICSAR145	4,785,805	163	64,178	4,575	4,512	69.32	ERS6092699	CAJSNV010000001
PICSAR147	4,789,937	175	68,132	4,576	4,513	69.32	ERS6092701	CAJSNO010000001
PICSAR148	4,788,670	141	79,766	4,561	4,498	69.32	ERS6092702	CAJSNM010000001
PICSAR15	4,780,402	214	49,347	4,581	4,518	69.31	ERS6092704	CAJSNY010000001
PICSAR154	4,789,709	162	70,010	4,586	4,523	69.32	ERS6092706	CAJSNL010000001
PICSAR157	4,787,040	166	62,990	4,573	4,510	69.32	ERS6092707	CAJSNN010000001
PICSAR16	4,789,808	170	64,202	4,578	4,515	69.32	ERS6092708	CAJSNK010000001
PICSAR160	4,787,812	173	66,624	4,566	4,503	69.32	ERS6092709	CAJSOD010000001
PICSAR162	4,785,890	195	49,541	4,584	4,521	69.31	ERS6377153	CAJSOA010000001
PICSAR164	4,769,542	325	30,069	4,608	4,545	69.29	ERS6092710	CAJSOC010000001
PICSAR168B	4,790,339	151	86,432	4,561	4,498	69.33	ERS6377154	CAJSOB010000001
PICSAR168	4,790,212	149	74,685	4,563	4,500	69.32	ERS6092711	CAJSNZ010000001
PICSAR172	4,788,168	144	74,172	4,569	4,506	69.32	ERS6092713	CAJSOE010000001
PICSAR178	4,773,707	183	58,645	4,571	4,508	69.32	ERS6092714	CAJSOH010000001
PICSAR179	4,788,184	192	59,069	4,587	4,524	69.32	ERS6092715	CAJSOK010000001
PICSAR18	4,783,336	218	47,690	4,594	4,532	69.31	ERS6092716	CAJSOM010000001
PICSAR180	4,790,473	144	79,784	4,568	4,505	69.32	ERS6092717	CAJSOG010000001
PICSAR181	4,783,299	213	48,895	4,583	4,520	69.31	ERS6092718	CAJSOI010000001
PICSAR182	4,786,048	199	53,495	4,576	4,513	69.32	ERS6092719	CAJSOF010000001
PICSAR184	4,789,623	168	66,614	4,575	4,512	69.32	ERS6092720	CAJSOJ010000001
PICSAR189	4,788,806	179	69,522	4,565	4,502	69.32	ERS6092721	CAJSOL010000001
PICSAR19	4,789,870	163	64,491	4,569	4,506	69.32	ERS6092722	CAJSOQ010000001
PICSAR191	4,789,697	173	67,710	4,564	4,501	69.32	ERS6092723	CAJSOR010000001
PICSAR192	4,788,235	193	58,604	4,595	4,532	69.31	ERS6092724	CAJSOU010000001
PICSAR196	4,791,434	161	71,932	4,570	4,507	69.32	ERS6092725	CAJSOS010000001
PICSAR197	4,791,144	133	85,113	4,570	4,507	69.33	ERS6092726	CAJSOO010000001
PICSAR198	4,787,281	151	74,155	4,563	4,500	69.32	ERS6092727	CAJSON010000001
PICSAR2	4,792,264	156	77,040	4,574	4,511	69.33	ERS6092728	CAJSOW010000001
PICSAR201	4,789,399	170	68,914	4,567	4,504	69.32	ERS6092730	CAJVAW010000001
PICSAR202	4,783,908	160	64,785	4,561	4,498	69.31	ERS6092731	CAJSOX010000001
PICSAR204	4,790,713	139	86,451	4,571	4,508	69.32	ERS6092733	CAJSOV010000001
PICSAR205	4,792,146	154	74,990	4,564	4,501	69.32	ERS6377155	CAJSOP010000001
PICSAR206	4,790,443	140	73,462	4,568	4,505	69.32	ERS6092734	CAJSOT010000001
PICSAR208	4,792,845	147	73,575	4,572	4,509	69.32	ERS6092735	CAJSPD010000001
PICSAR209	4,785,508	185	62,895	4,579	4,516	69.32	ERS6092736	CAJSPG010000001
PICSAR21	4,795,268	165	71,391	4,579	4,516	69.32	ERS6377156	CAJSPA010000001
PICSAR210	4,786,061	170	64,188	4,581	4,518	69.32	ERS6092737	CAJVRD010000001
PICSAR211	4,788,728	133	83,580	4,551	4,488	69.33	ERS6092738	CAJSPB010000001
PICSAR214	4,765,345	213	56,214	4,569	4,506	69.31	ERS6092740	CAJSPC010000001
PICSAR215	4,786,273	194	53,502	4,585	4,522	69.31	ERS6092741	CAJVAT010000001
PICSAR216	4,791,256	124	97,349	4,566	4,503	69.33	ERS6092742	CAJVAU010000001
PICSAR218	4,789,693	123	92,477	4,567	4,504	69.33	ERS6092743	CAJVAV010000001
PICSAR22	4,791,855	151	79,764	4,557	4,494	69.33	ERS6092746	CAJSPF010000001
PICSAR222	4,789,088	183	60,343	4,588	4,525	69.32	ERS6092747	CAJSPE010000001
PICSAR23	4,787,284	142	92,228	4,566	4,503	69.32	ERS6092749	CAJSPH010000001
PICSAR232	4,790,364	144	73,122	4,563	4,500	69.32	ERS6092750	CAJSPO010000001
PICSAR235	4,781,829	221	43,461	4,598	4,535	69.31	ERS6092751	CAJSPQ010000001
PICSAR238	4,788,942	173	64,108	4,582	4,520	69.32	ERS6092752	CAJSPP010000001
PICSAR24	4,786,946	159	77,065	4,563	4,500	69.32	ERS6092753	CAJSPK010000001
PICSAR240	4,779,977	168	62,880	4,571	4,508	69.31	ERS6092754	CAJSPN010000001
PICSAR246	4,787,832	195	58,821	4,591	4,528	69.32	ERS6092756	CAJSPR010000001
PICSAR248	4,790,004	160	69,396	4,586	4,523	69.32	ERS6092758	CAJSPJ010000001
PICSAR249	4,792,479	176	58,262	4,581	4,518	69.32	ERS6092759	CAJSPL010000001
PICSAR25	4,781,362	211	48,503	4,578	4,517	69.31	ERS6377157	CAJSPM010000001
PICSAR250	4,792,163	144	86,432	4,576	4,513	69.32	ERS6092760	CAJSPI010000001
PICSAR252	4,786,258	187	58,262	4,591	4,528	69.32	ERS6092762	CAJSPS010000001
PICSAR253	4,794,805	163	77,033	4,571	4,508	69.31	ERS6377158	CAJSPT010000001
PICSAR254	4,790,393	167	69,690	4,577	4,514	69.33	ERS6092763	CAJSPV010000001
PICSAR255	4,789,281	155	71,960	4,572	4,509	69.33	ERS6092764	CAJSPW010000001
PICSAR26	4,803,390	358	31,565	4,642	4,579	69.3	ERS6092767	CAJSPY010000001
PICSAR28	4,784,222	178	58,791	4,577	4,514	69.31	ERS6092769	CAJSPX010000001
PICSAR29	4,790,559	180	55,537	4,586	4,523	69.32	ERS6092770	CAJSPU010000001
PICSAR3	4,788,567	149	86,432	4,566	4,503	69.33	ERS6092771	CAJSPZ010000001
PICSAR30	4,788,254	144	73,587	4,558	4,495	69.32	ERS6092772	CAJSQA010000001
PICSAR36	4,788,733	173	64,182	4,578	4,515	69.32	ERS6092773	CAJSQH010000001
PICSAR38	4,783,227	188	56,621	4,589	4,526	69.31	ERS6092774	CAJSQB010000001
PICSAR4	4,789,442	161	69,522	4,572	4,509	69.32	ERS6092775	CAJSQE010000001
PICSAR43	4,795,124	164	74,414	4,570	4,507	69.32	ERS6092776	CAJSQC010000001
PICSAR46	4,789,901	187	60,696	4,595	4,532	69.32	ERS6092777	CAJSQI010000001
PICSAR47	4,788,818	150	71,401	4,572	4,509	69.32	ERS6092778	CAJSQD010000001
PICSAR48	4,785,510	199	49,332	4,578	4,515	69.31	ERS6092779	CAJSQG010000001
PICSAR49	4,781,084	182	62,918	4,585	4,522	69.31	ERS6092780	CAJSQF010000001
PICSAR5	4,784,540	230	42,694	4,594	4,531	69.31	ERS6092781	CAJSQJ010000001
PICSAR51	4,793,239	189	59,822	4,591	4,528	69.32	ERS6092782	CAJSQL010000001
PICSAR52	4,787,441	152	72,686	4,573	4,510	69.32	ERS6092783	CAJSQM010000001
PICSAR53	4,784,438	179	58,265	4,598	4,535	69.31	ERS6092784	CAJSQN010000001
PICSAR55	4,785,207	218	40,946	4,606	4,543	69.31	ERS6092785	CAJSQK010000001
PICSAR56	4,788,148	166	68,167	4,578	4,515	69.32	ERS6092786	CAJSQP010000001
PICSAR58	4,787,822	178	64,942	4,567	4,504	69.32	ERS6092787	CAJSQO010000001
PICSAR6	4,787,390	196	56,620	4,601	4,538	69.32	ERS6092789	CAJSQQ010000001
PICSAR61	4,787,489	180	59,708	4,572	4,509	69.32	ERS6092791	CAJSQR010000001
PICSAR63	4,790,650	162	71,378	4,572	4,509	69.32	ERS6092792	CAJSQS010000001
PICSAR64	4,787,452	193	58,783	4,580	4,517	69.31	ERS6092793	CAJSQT010000001
PICSAR65	4,782,888	305	30,419	4,639	4,576	69.3	ERS6092794	CAJSQU010000001
PICSAR68	4,786,445	181	58,792	4,565	4,502	69.32	ERS6092796	CAJSQZ010000001
PICSAR7	4,778,202	248	36,939	4,597	4,534	69.3	ERS6092797	CAJSQV010000001
PICSAR70	4,784,997	142	92,177	4,585	4,522	69.32	ERS6092798	CAJSRA010000001
PICSAR71	4,781,870	222	48,298	4,620	4,557	69.31	ERS6092799	CAJSQX010000001
PICSAR72	4,788,047	175	59,133	4,574	4,511	69.32	ERS6092800	CAJSRC010000001
PICSAR73	4,785,412	174	64,178	4,573	4,510	69.32	ERS6092801	CAJSQY010000001
PICSAR74	4,793,447	159	66,779	4,569	4,506	69.32	ERS6092802	CAJSRB010000001
PICSAR75	4,789,467	174	66,593	4,569	4,506	69.32	ERS6092803	CAJSRD010000001
PICSAR76	4,791,598	148	82,825	4,567	4,504	69.32	ERS6092804	CAJSQW010000001
PICSAR77	4,792,094	147	86,451	4,565	4,502	69.32	ERS6092805	CAJSRF010000001
PICSAR78	4,787,636	205	53,988	4,592	4,529	69.31	ERS6092806	CAJSRG010000001
PICSAR8	4,782,208	192	49,547	4,565	4,502	69.31	ERS6092807	CAJSRE010000001
PICSAR81	4,787,134	187	58,883	4,563	4,500	69.32	ERS6092808	CAJSRH010000001
PICSAR85	4,785,784	170	64,173	4,577	4,514	69.32	ERS6377159	CAJSRJ010000001
PICSAR9	4,791,470	127	85,615	4,566	4,503	69.33	ERS6092809	CAJSRI010000001
PICSAR94	4,788,723	179	64,188	4,578	4,515	69.32	ERS6092810	CAJSRK010000001
PICSAR98	4,791,377	137	85,433	4,563	4,500	69.32	ERS6092811	CAJSRL010000001
PICSAR42	4,779,801	229	50,081	4,588	4,526	69.3	ERS6377160	CAJSRM010000001
PICSAR39	4,782,076	212	47,711	4,581	4,518	69.31	ERS6377161	CAJSRN010000001
PICSAR138	4,783,258	239	40,551	4,590	4,527	69.3	ERS6377162	CAJSRO010000001
PICSAR97	4,784,339	215	46,530	4,596	4,533	69.31	ERS6377163	CAJSRP010000001
PICSAR213	4,788,192	152	80,210	4,568	4,505	69.32	ERS6377164	CAJSRR010000001
PICSAR87	4,797,579	167	66,614	4,567	4,504	69.3	ERS6377165	CAJSRQ010000001
PICSAR171	4,790,264	162	65,791	4,567	4,504	69.31	ERS6377166	CAJSRX010000001
PICSAR200	4,794,348	148	86,432	4,572	4,509	69.32	ERS6377167	CAJSRW010000001
PICSAR236	4,790,200	147	79,765	4,560	4,497	69.33	ERS6377168	CAJSRS010000001
PICSAR35	4,780,919	218	46,619	4,584	4,521	69.31	ERS6377169	CAJSRV010000001
PICSAR190	4,788,050	202	52,943	4,584	4,521	69.3	ERS6377170	CAJSRT010000001
PICSAR144	4,781,205	174	71,974	4,575	4,512	69.32	ERS6092698	CAJSRU010000001

a CDSs, coding DNA sequences.

### Data availability.

The sequences were deposited in the European Nucleotide Archive public sequence database under the accession numbers SAMEA8407198 to SAMEA8407344 and SAMEA8692664 to SAMEA8692685 (BioProject accession number PRJEB43898).
